# Highly Conductive PEDOT:PSS Transparent Hole Transporting Layer with Solvent Treatment for High Performance Silicon/Organic Hybrid Solar Cells

**DOI:** 10.1186/s11671-017-2276-5

**Published:** 2017-08-23

**Authors:** Qingduan Li, Jianwei Yang, Shuangshuang Chen, Jizhao Zou, Weiguang Xie, Xierong Zeng

**Affiliations:** 1 Shenzhen Key Laboratory of Special Functional Materials and Shenzhen Engineering Laboratory for Advance Technology of Ceramics, College of Materials Science and Engineering, Shenzhen University, Shenzhen, 518060 People’s Republic of China; 2 Key Laboratory of Optoelectronic Devices and Systems of Ministry of Education and Guangdong Province, College of Optoelectronic Engineering, Shenzhen University, Shenzhen, 518060 People’s Republic of China; 3 Department of Physics and Department of Electronic Engineering, Siyuan Laboratory, Guangzhou Key Laboratory of Vacuum Coating Technologies and New Energy Materials, Jinan University, Guangzhou, 510632 China

**Keywords:** Si/organic, Hybrid solar cells, PEDOT:PSS, Treatment, Conductivity

## Abstract

Efficient Si/organic hybrid solar cells were fabricated with dimethyl sulfoxide (DMSO) and surfactant-doped poly(3,4-ethylenedioxythiophene): polystyrene (PEDOT:PSS). A post-treatment on PEDOT:PSS films with polar solvent was performed to increase the device performance. We found that the performance of hybrid solar cells increase with the polarity of solvent. A high conductivity of 1105 S cm^− 1^ of PEDOT:PSS was achieved by adopting methanol treatment, and the best efficiency of corresponding hybrid solar cells reaches 12.22%. X-ray photoelectron spectroscopy (XPS) and RAMAN spectroscopy were utilized to conform to component changes of PEDOT:PSS films after solvent treatment. It was found that the removal of the insulator PSS from the film and the conformational changes are the determinants for the device performance enhancement. Electrochemical impedance spectroscopy (EIS) was used to investigate the recombination resistance and capacitance of methanol-treated and untreated hybrid solar cells, indicating that methanol-treated devices had a larger recombination resistance and capacitance. Our findings bring a simple and efficient way for improving the performance of hybrid solar cell.

**Electronic supplementary material:**

The online version of this article (doi:10.1186/s11671-017-2276-5) contains supplementary material, which is available to authorized users.

## Background

In recent years, silicon-organic hybrid solar cells are attracting great attention benefit from their advantages such as low-temperature spin-coating process, simple device structure, and low-cost potential [1–7]. Several kinds of organic materials, including conjugated polymers [1–4, 8], conjugated small molecules [9, 10], and fullerene derivatives [11], are used as hole or electron transporting layer in hybrid solar cells. Among them, poly(3,4-ethylenedioxythiophene): polystyrene (PEDOT:PSS), a conducting polymer widely used as a hole transporting layer or metal-free electrode in organic electronic devices, has been proven to be commendable to act as a hole transporting layer in hybrid solar cells [12–15]. Owing to the rapid development of theory and techniques on high-performance materials [16, 17], hybrid solar cells have gained great progress. Generally, in a Si/PEDOT:PSS heterojunction-based solar device, the incoming light is mostly absorbed by Si. Light-induced charge carriers are then separated under the built-in electric field. In order to get high-power conversion efficiency hybrid solar cells, many efforts have been made to reduce the light reflection of the Si substrate. Therefore, nanostructured Si including nanowires [1], nanoholes [18], pyramids [19], and some other hierarchical structures [20] are applied to increase the light harvesting of the hybrid solar cells. Although an enhanced short-circuit current intensity (*J*_SC_) may be obtained due to the improved light harvesting, the associated large surface/volume ratio of nanostructured Si may cause poor contact between Si and PEDOT:PSS and then serious surface recombination in the hybrid solar cells. What is more, the cost will be increased with complex nanostructure Si fabrication. On the other hand, it has been reported that the conductivity and the contact between PEDOT:PSS and Si could be improved by adding organic co-solvents and non-ionic surfactant, respectively. It has been reported that improvement of surface conductivity of PEDOT:PSS films could been received by acid treatments like formic acid treatment and nitric acid treatment [21, 22]. But acid treatment is too violent for the PEDOT:PSS films and may take adverse effects to the device stability. It is well known that PEDOT:PSS aqueous dispersion is made up of a certain concentration of PSS added to PEDOT. But the insulating PSS that contains sulfonic acid SO_3_H groups may bring detrimental effects such as low conductivity and lifetime issues. Dimethyl sulfoxide (DMSO) and ethylene glycol (EG) are commonly used as co-solvents to modify the morphology and nanostructure of PEDOT:PSS, and the conductivity could be significantly improved compared to that with other co-solvents [23, 24]. However, it is worth noting that although the morphological structure across the PEDOT:PSS thin film may be modified by the addition of co-solvents, the negative effects brought by PSS still remain, which means the performance of the hybrid solar cells could be further improved.

In this work, we demonstrate planar Si-based hybrid solar cells with an enhanced PCE by a simple post-treatment with methanol. DMSO is used as a co-solvent to improve the conductivity of the PEDOT:PSS thin film; in addition, a further methanol treatment by spin-coating could further improve the conductivity and change the PSS concentration on the surface. A high PCE of 12.22% has been achieved by the methanol-treated hybrid Si/PEDOT:PSS solar cell, which is 28% higher than that of the untreated one. The effects of surface treatment with different alcohols on the hybrid solar cells properties are evaluated. Our work offers a better understanding of using of solvent treatment for further enhancing the device performances of the hybrid Si/organic solar cells. Our experimental results demonstrate that an effective modification of electrical properties occurs in Si/PEDOT:PSS solar cells when implementing methanol treatment on PEDOT:PSS films.

## Methods

Double-side-polished n-type CZ crystal Si(100) wafers(2.6 ~ 3.5 Ω cm, 450-μm thickness) were first cleaned using acetone, ethanol, and deionized water by ultrasonically soaking for 20 min, respectively. Then, the substrates were treated in a 80 °C piranha solution (3:1 H_2_SO_4_/H_2_O_2_) for 30 min and washed with deionized water several times. Finally, the samples were immersed in a diluted HF (5%) solution for 5 min to remove the native oxide to obtain H-Si surfaces. The cleaned Si were then transferred into a diluted HNO_3_ (10%) solution to form a SiO_*x*_ film to act as a passivation layer [25, 26]. Highly conductive PEDOT:PSS (Clevios PH1000) uniformly mixed with 5 wt% DMSO and 1 wt% Triton X-100 was spin-coated onto the surface of the SiO_*x*_-terminated Si substrate at a spin speed of 1500 rpm in air for 60 s. Following that, the samples were annealed at 140 °C for 10 min under nitrogen atmosphere. Solvent treatment with methanol or other alcohols on PEDOT:PSS films was done by dropping 60 μL methanol or other alcohols on the dried PEDOT:PSS films and then spin-coated at 2000 rpm for 60 s. The obtained films were annealed at 120 °C for 10 min under nitrogen atmosphere. Silver grids of 200-nm thickness were deposited by thermal evaporation as the top electrode through a shadow mask and aluminum of 200-nm thickness was deposited on the back side. The deposition process is performed under high vacuum circumstance about ~ 10^− 7^ Pa. The deposition rate of Ag is controlled at 0.2 Ȧ S^− 1^ for the first 10 nm and at 0.5 Ȧ S^− 1^ for the rest of the Ag electrode. And for Al deposition, the deposition rate is controlled at 0.3 Ȧ S^− 1^ for the first 10 nm, 1 Ȧ S^− 1^ for the thickness range from 10 to 200 nm, and 5 Ȧ S^− 1^ for the rest part. The device area is 0.3 cm^2^.

The current density-voltage (*J-V*) characteristics of the solar cells were determined by a Keithley 2400 digital source meter under simulated sunlight (100 mW cm^− 2^) illumination provided by a xenon lamp (Oriel) with an AM 1.5 filter. The radiation intensity was calibrated by a standard silicon photovoltaic device. The external quantum efficiency (EQE) system used a 300 W xenon light source with a spot size of 1 mm × 3 mm which was calibrated with a silicon photodetector. For PEDOT:PSS conductivity measurements, the PEDOT:PSS films are spin-coating on a glass. The conductivity of the PEDOT:PSS films was measured by RST-9 4-point probe instrument. The X-ray photoelectron spectroscopy (XPS) spectra were collected on Thermo ESCALAB 250 equipped with a monochromatized Al Kα source (*hν* = 1486.8 eV). Electrochemical impedance spectroscopy (EIS) was performed using an electrochemical workstation (CHI660E). EIS spectra are recorded in the frequency range of 10^− 1^–10^6^ Hz at room temperature. The results of EIS spectra are analyzed and fitted using the *Z*-view software. Transmittance spectra of the films were measured using a UV-2450 spectrophotometer with PEDOT:PSS films spin-coating on a quartz glass. The surface topography and roughness of PEDOT:PSS films were observed by atomic force microscopy (AFM) in a Digital Instruments Dimension 3100 Nanoscope IV.

## Results and Discussion

### PEDOT:PSS/Planar-Si Hybrid Solar Cell Properties

**Scheme 1:**
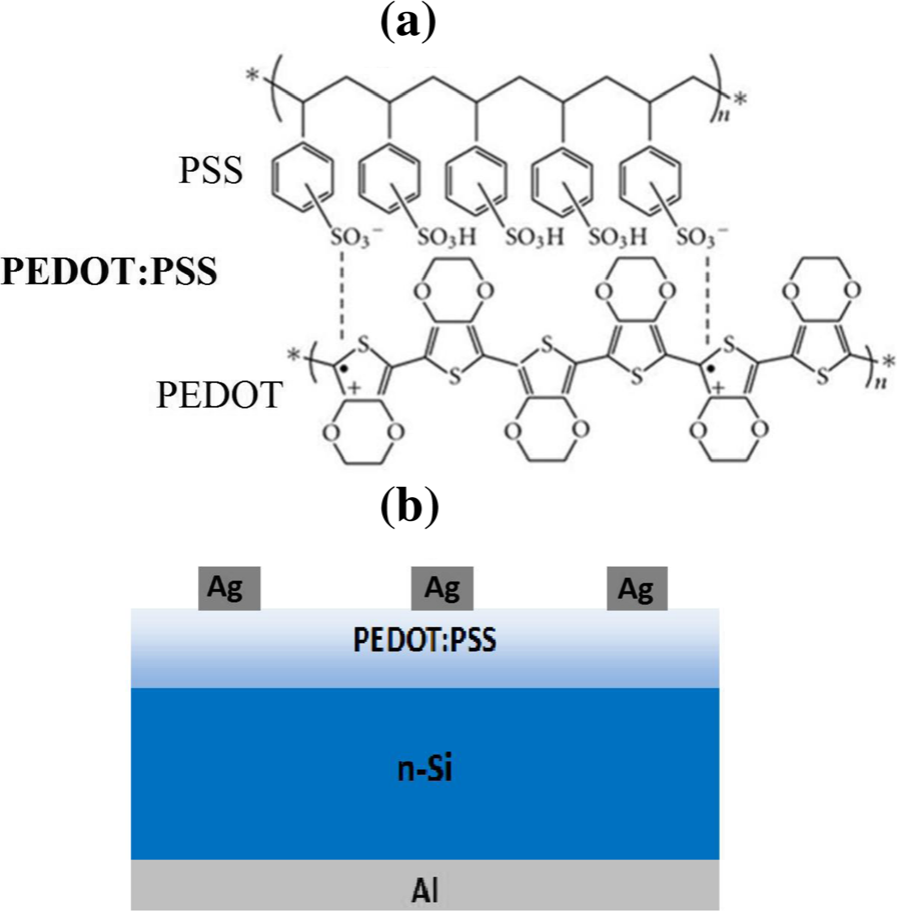
**a** Molecule structure of PEDOT:PSS. **b** Device structure

**Fig. 1:**
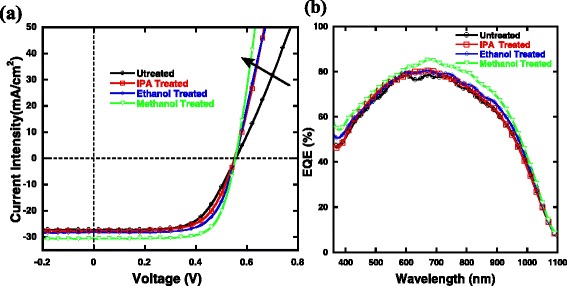
**a** *J-V* curves under the illumination of AM 1.5, 100 mW cm^− 2^, and **b** corresponding EQE spectra

Scheme 1 presents the molecule structure of PEDOT:PSS and the device structure of planar Si/organic solar cells. Figure 1 shows the light current *J-V* and EQE spectra curves of hybrid solar cells treated with different alcohols, and the solar cell parameters, including *J*_SC_, *V*_OC_, *FF*, and PCE, are summarized in Table 1. The average solar cell performance is calculated based on more than ten devices. The control device with DMSO as co-solvents without post-treatment shows a *V*_OC_ of 0.552 V, a *J*_SC_ of 27.09 mA cm^− 1^, and a *FF* of 63.60%, leading to a PCE of 9.51%. To examine the effect of post-treatment on the device performance, different solvents, i.e., IPA, ethanol, and methanol, with increasing polarity were selected to modify the PEDOT:PSS. The physical properties of IPA, ethanol, and methanol are summarized in Table 2 [27].

**Table 1:** Photovoltaic performance of the hybrid solar cells with PEDOT:PSS treated with different chemicals

Treatment	PCE_max_(PCE_ave_)^a^ (%)	*V*_OC_ (V)	*FF* (%)	*J*_SC_(mA cm^− 2^)	*J*_SC_ (cal)^b^(mA cm^−2^)
Untreated	9.51 (9.35)	0.552	63.14	27.09	26.68
IPA	9.98 (9.62)	0.557	64.66	27.71	27.35
Ethanol	10.69 (10.41)	0.556	68.27	28.16	27.74
Methanol	12.22 (12.13)	0.555	72.01	30.58	30.11

^a^The average PCE was obtained from more than 10 devices

^b^ *J*_SC_ calculated photocurrent density from EQE measurements

**Table 2:** Physical properties of solvents used for film treatments

Chemical	Boiling pt./°C	Dielectric constant	Absolute viscosity	Polarity (water = 100)
IPA	82	18.3	2.0	54.6
Ethanol	78	22.2	1.08	65.4
Methanol	64	32.6	0.6	76.2

Compared to untreated devices, a slightly higher PCE of 9.98% is achieved for IPA-treated devices, with a *J*_SC_ of 27.71 mA cm^− 1^ and a *FF* of 64.66%. The ethanol-treated devices have a *V*_OC_ of 0.556 V, a *J*_SC_ of 28.16 mA cm^− 1^, and a *FF* of 68.27%, resulting in a higher PCE of 10.69%. When methanol treatment was used, a highest PCE of 12.22% is achieved with a *J*_SC_ of 30.58 mA cm^− 1^ and a *FF* of 72.01%, which is 28% higher than that of control devices. Obviously, the performance of hybrid solar cells is increased with increasing polarities of chemicals used.

### Conductivity and Optoelectronic Properties of Treated PEDOT:PSS Films

**Fig. 2:**
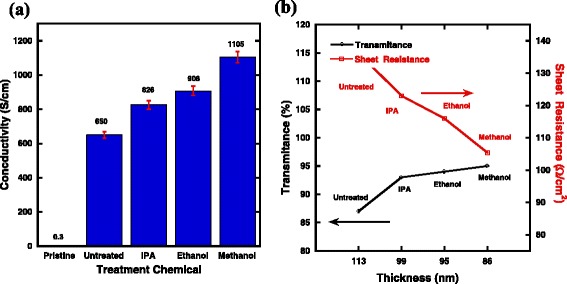
**a** Conductivities of PEDOT:PSS films treated with different chemicals. **b** Variation of transmittance and sheet resistance for PEDOT:PSS treated with different chemicals

In order to understand the influence of solvent treatment on device performance of hybrid solar cells, the conductivity was measured by a 4-point probe instrument. Transmittance spectra of the films were also measured using spectrophotometer. Conductivity values along with the error bars of pristine PEDOT:PSS films and after film treatment with different alcohols are shown in Fig. 2a. Conductivity of PEDOT:PSS films without DMSO as additive solvent were also measured here. It can be seen from Fig. 2a that the average conductivity drastically increases from 0.3 to 650 S cm^− 1^with DMSO as additive solvent. As it can be clearly seen in Fig. 2a and Table 2, the conductivity increases with increasing dielectric constants and polarities of the alcohols. Given this tendency, the average conductivities for PEDOT:PSS films with a further treatment with IPA and ethanol are 826 and 908 S cm^− 1^, respectively. For methanol-treated films, an average conductivity of 11 S cm^− 1^ is achieved. It is much higher than the reported value [23]. It is well known that the Coulomb interaction between positively charged PEDOT and negatively charged PSS dopants could be reduced by polar solvents [28]. So, higher dielectric constant of the polar solvent will lead to a stronger screening effect between counter ions and charge carriers during the treatment process. As a result, the thickness of treated PEDOT:PSS varies with different treating chemicals. Figure 2b shows the variation of sheet resistance and transmittance at 550 nm of the PEDOT:PSS films treated with different alcohols. As shown by the *X*-axis of Fig. 2b, the thicknesses are 113, 99, 95, and 86 nm for untreated, IPA-treated, ethanol-treated, and methanol-treated films, respectively. The methanol-treated films show a sheet resistance of 105 Ω cm^−  2^ and a transmittance of 95%. Films treated with different alcohols have almost equal transmittance value, indicating that the film treatment mainly affects the electronic properties of the PEDOT:PSS films.

**Fig. 3:**
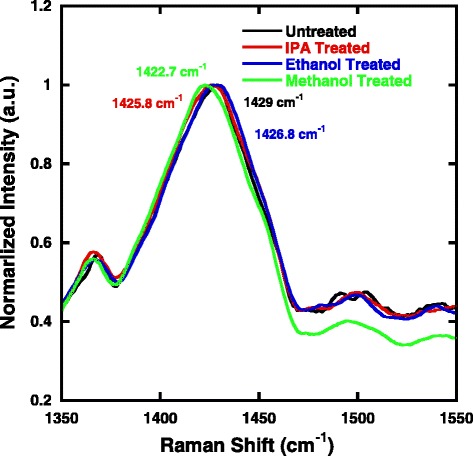
Raman spectra of the untreated PEDOT:PSS film and the PEDOT:PSS films treated with different chemicals

**Scheme 2:**
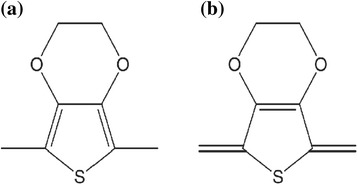
**a** Benzoid and **b** quinoid structures exist within PEDOT

It has been shown that the reorganization of PEDOT nanocrystals in the spin-coating PEDOT:PSS thin films can be identified by Raman spectroscopy [29]. We thus carried out Raman measurements to investigate the difference between the treated and untreated PEDOT:PSS films. Figure 3 shows the Raman spectra of the PEDOT:PSS films treated with different methods. In the chemical structure of PEDOT, there are two resonant structures, namely, benzoid and quinoid as depicted in Scheme 2 [30]. In the benzoid structure, the C_α_–C_β_ bond is formed by two conjugated electrons, while in the quinoid structure, there are no conjugated *π*-electrons on the C_α_–C_β_ bond. The quinoid structure shows more rigidity than the benzoid structure. The rigid quinoid structure has more strong interactions among the PEDOT chains leading to high charge carrier mobility. As shown in Fig. 3, for ethanol- and IPA-treated films, the shifts are from 1429 to 1426.8 cm^− 1^ and 1429 to 1425.8 cm^− 1^, respectively, compared to untreated films. And the methanol-treated PEDOT:PSS film shows a shift from 1429 to 1422.7 cm^− 1^ compared to the untreated PEDOT:PSS film. The increasing Raman shift is consistent with the increasing of polarity, and it indicates that the methanol treatment promotes the most conformation changes from benzoid to quinoid structure [30]. In other words, methanol treatment is the most effective way to remove insulating PSS component in PEDOT:PSS film and promote more rigid structure and packing of PEDOT chains, leading to an enhanced performance.

**Fig. 4:**
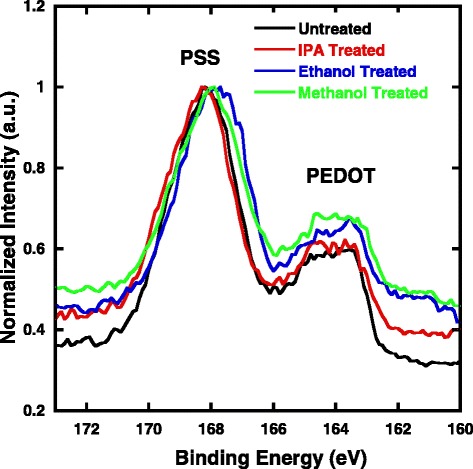
S (2p) XPS spectra of untreated and methanol-treated PEDOT:PSS films

To further understand whether the PSS matrix on the surface of the PEDT:PSS film are to some extent removed after solvent treatment, XPS experiments are performed to explore component changes of the PEDOT:PSS film after spin-coating treatment. Figure 4 shows XPS spectra of the S2p of PEDOT:PSS films prepared with/without post-treatment with different alcohols. The band between 166 and 172 eV corresponds to the sulfur atom in PSS, and the band between 162 and 166 eV corresponds to the sulfur atoms in PEDOT [31, 32]. The ratio of band areas for PSS to PEDOT can be used to calculate the relative composition of PSS to PEDOT at the surface. The summary of peak areas of the amount of PSS to that of PEDOT at the surface is listed in Additional file 1: Table S1. The untreated PEDOT:PSS film shows a PSS/PEDOT ratio of 2.48, which is in accordance to the already accepted conclusion that the surface of a PEDOT:PSS film contains more PSS than that in the bulk [33]. For ethanol- and IPA-treated films, the PSS/PEDOT ratio is 1.50 and 1.87, indicating that a certain extent insulating PSS was washed off during the solvent treatment. For the films with methanol treatment, the PSS/PEDOT ratio is decreased to 1.33. The trend of the decreased PSS/PEDOT ratio is consistent to the increased electrical conductivity of the resultant PEDOT:PSS films. We also carried out AFM studies to investigate the influence of methanol treating on surface structure. Through the height images in Additional file 1: Figure S1, both treated and untreated PEDOT:PSS films have highly smooth surface characteristics. Nanofibril-like structures could be found in both films, which could be attributed to the effect of pre-adding DMSO. AFM measurements indicate that there is no distinct change on chain structure of PEDOT:PSS. The surface roughness estimated from AFM for the untreated PEDOT:PSS film is 2.08 nm and 2.38 nm for the treated film.

**Fig. 5:**
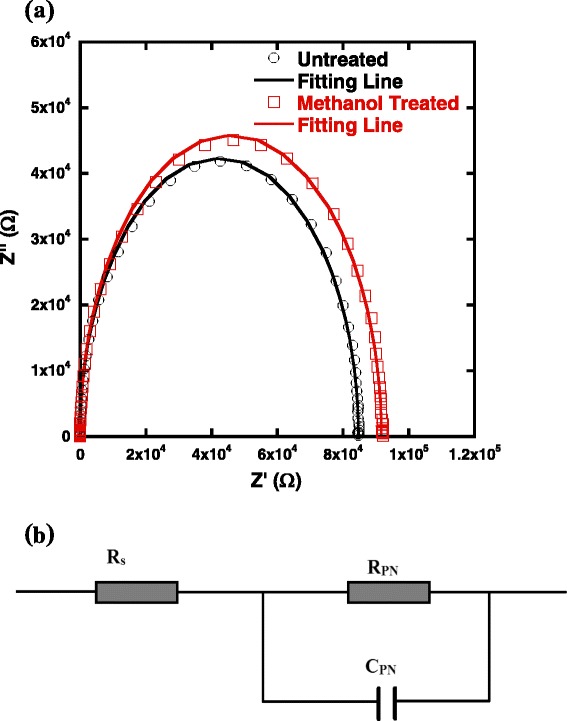
**a** EIS (Nyquist plots) of untreated and methanol-treated Si/PEDOT:PSS hybrid solar cells under zero bias voltage, experimental data are represented by dots, and fitting data according to the relevant models are represented by lines, respectively. **b** Equivalent circuit model to fit the experimental data

Impedance spectroscopy measurement is a powerful technique to probe the physical processes, such as carrier transfer and recombination at internal interfaces, using a proper RC element [34, 35]. Mott-Schottky (MS) curves were also measured for methanol-treated and untreated hybrid solar cells. According to Anderson’s model, the capacitance is described by the following equation [36].

1 $$ {C}^{-2}=\frac{V_{\mathrm{bi}}-{V}_{\mathrm{app}}}{A^2q{\varepsilon}_0{\varepsilon}_{\mathrm{r}}{N}_{\mathrm{A}}}, $$

where *V*_bi_ is the built-in voltage, *V*_app_ is the applied voltage, *ɛ*_r_ is the relative dielectric constant, *ε*_0_ is the vacuum permittivity, and *N*_A_ is the acceptor impurity concentration. The 1/*C* ^2^-*V* plots of the hybrid solar cells are shown in Additional file 1: Figure S2; the extrapolated intercept in the potential coordinate axis indicated that methanol treatment does not show an ambiguous impact on the built-in potential. The Nyquist plots of hybrid solar cells measured under open-circuit condition are shown in Fig. 5a. The sole semicircle observed in each plot indicates only a RC element at the interface of the Si/PEDOT:PSS heterojunction, and the equivalent circuit is presented in Fig. 5b. According to diffusion-reaction model [37], the arc impedance of this circuit is given by

2 $$ Z\left(\upomega \right)={Z}^{\prime}\left(\upomega \right)-\mathrm{j}\left(\upomega \right){Z}^{{\prime\prime} }, $$

where *Z*′and *Z*″are the magnitudes of the real and imaginary parts of impedance, and a minus sign arises due to the capacitive reactance involved in the circuit. The fitted curves match well with the experimental data, suggesting that the circuit model reflects the real circuit. The resistance element *R*_PN_ and the capacitance element *C*_PN_ are estimated from the fitting data. The minority carrier lifetime (*τ*) at the related interfaces of hybrid solar cells could be determined by *τ* = *R*_PN_ × *C*_PN_ [38]. The fitting parameters are compared in Additional file 1: Table S2. R_PN_ is a critical factor for device performance because a high *R*_PN_ implies reduced carrier loss through recombination. As shown in Additional file 1: Table S2, a longer carrier lifetime is obtained for methanol-treated devices (751.12 μs) than that for untreated devices (621.81 μs) under open-circuit condition, suggesting more effectively electron blocking at PEDOT:PSS/Ag interface in methanol-treated devices.

## Conclusions

In summary, a post-treatment on PEDOT:PSS films with polar solvent has been proposed to enhance the performance of PEDOT:PSS/Si heterojunction solar cells. A high conductivity of 1105 S cm^− 1^ of PEDOT:PSS was achieved by using methanol treatment as the corresponding hybrid solar cells having a best efficiency of 12.22%, which is 28% higher compared to those with untreated PEDOT:PSS films. RAMAN and XPS results provide strong evidence for the reorganization of PEDOT nanocrystals and reduction of PSS chain along the surface, which jointly enhance the conductivity and therefore the device performance. The enhanced conductivity can be ascribed to the rearrangement of PEDOT moieties on the surface since the PSS matrix can be removed by methanol spin-coating. EIS measurements stated clearly results that the charge recombination loss in hybrid solar cells with methanol treated PEDOT:PSS films is reduced compared to untreated devices. We believe that such low cost approaches of modifying the surface of PEDOT:PSS buffer layer would be promising candidates for photovoltaic application.

## Additional file

Additional file 1: Table S1. The summary of peak areas of the amount of PSS to that of PEDOT at the surface (as estimated by the ratio of the respective S2p3/2 peak areas). **Figure S1.** Topographic AFM images of (a) the untreated PEDOT:PSS film and (b) methanol-treated PEDOT:PSS film. **Table S2.** Parameters employed for the fitting of the impedance spectra. **Figure S2.** *C* ^− 2^-*V* plot of untreated and methanol-treated hybrid devices; experimental data are represented by dots, and the fit linear data are represented by a line. (DOCX 420 kb)
