# Supplementary material for: Highly Conductive PEDOT:PSS Transparent Hole Transporting Layer with Solvent Treatment for High Performance Silicon/Organic Hybrid Solar Cells
Source: Nanoscale Res Lett. 2017 Aug 23;12:506. doi: 10.1186/s11671-017-2276-5 (PMC6890909; doi:10.1186/s11671-017-2276-5)
Supplement: Supplementary file 1 — The summary of peak areas of the amount of PSS to that of PEDOT at the surface (as estimated by the ratio of the respective S2p3/2 peak areas). Figure S1. Topographic AFM images of (a) the untreated PEDOT:PSS film and (b) methanol-treated PEDOT:PSS film. Table S2. Parameters employed for the fitting of the impedance spectra. Figure S2. C − 2-V plot of untreated and methanol-treated hybrid devices; experimental data are represented by dots, and the fit linear data are represented by a line. (DOCX 420 kb) [file 11671_2017_2276_MOESM1_ESM.docx]

**Additional file for**

**Highly Conductive PEDOT:PSS Transparent Hole Transporting Layer with Solvent Treatment for** **High Performance Silicon/Organic Hybrid Solar Cells**

Qingduan Li^a,^^b^, Jianwei Yang^a^, Shuangshuang Chen^a,b^, JizhaoZou^a^*,WeiguangXie^c^*,XierongZeng^a^

^a^Shenzhen Key Laboratory of Special Functional Materials & Shenzhen Engineering Laboratory for Advance Technology of Ceramics, College of Materials Science and Engineering, Shenzhen University, Shenzhen 518060, PR China. E-mail: [zoujizhao@szu.edu.cn](mailto:zoujizhao@szu.edu.cn)

^b^Key Laboratory of Optoelectronic Devices and Systems of Ministry of Education and Guangdong Province, College of Optoelectronic Engineering, Shenzhen University, Shenzhen 518060, PR China

^c^Siyuan Laboratory, Guangzhou Key Laboratory of Vacuum Coating Technologies and New Energy Materials, Department of Physics and Department of Electronic Engineering, Jinan University, Guangzhou 510632, China. E-mail: [wgxie@email.jnu.edu.cn](mailto:wgxie@email.jnu.edu.cn)

**Table S1** The summary of peak areas of the amount of PSS to that of PEDOT at the surface (as estimated by the ratio of the respective S 2p3/2 peak areas)

| Treatment | PSS peak area | PEDOT peak area | PSS/PEDOT |
| --- | --- | --- | --- |
| Untreated | 2.29 | 0.93 | 2.46:1 |
| IPA | 2.15 | 1.15 | 1.87:1 |
| Ethanol | 2.14 | 1.42 | 1.50:1 |
| Methanol | 2.18 | 1.64 | 1.33.:1 |


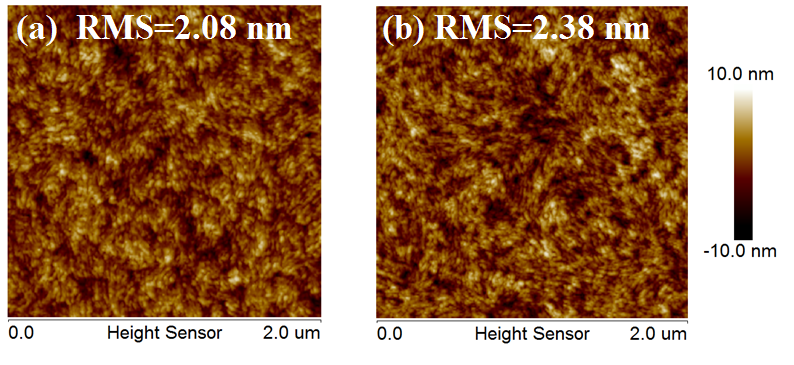


**FigureS1.**Topographic AFM images of (a) the untreated PEDOT:PSS ﬁlmand (b) methanol treated PEDOT:PSS film

**Table S2**. Parameters employed for the fitting of the impedance spectra.

| Methanol Treatment | R_PN_ (Ω) | C_PN_(F) | τ(μs) |
| --- | --- | --- | --- |
| Untreated | 8.46x10^4^ | 7.35x10^-9^ | 621.81 |
| Treated | 9.16x10^4^ | 8.20x10^-9^ | 751.12 |

**Figure S2**. C^-2^-V plot of untreated and methanol treated hybrid devices; experimental data arerepresented by dots and the fit linear data are represented by a line.
